# Nursing care directed to burned patients: a scoping review

**DOI:** 10.1590/0034-7167-2022-0205

**Published:** 2023-06-26

**Authors:** Pâmela Cristine Piltz Costa, Camila Schirmer Barbosa, Cristiano de Oliveira Ribeiro, Luana Aparecida Alves da Silva, Luciana de Alcantara Nogueira, Luciana Puchalski Kalinke

**Affiliations:** I Universidade Federal do Paraná. Porto Velho, Rondônia, Brazil; II Universidade Federal do Paraná. Curitiba, Paraná, Brazil

**Keywords:** Nursing, Burns, Wounds and Injuries, Nursing Care, Review, Enfermería, Quemaduras, Heridas y Lesiones, Atención de Enfermería, Revisión, Enfermagem, Queimaduras, Ferimentos e Lesões, Cuidados de Enfermagem, Revisão

## Abstract

**Objectives:**

to identify in the literature and summarize nursing care in a hospital environment directed to patients who suffered burns.

**Methods:**

a scoping review, according to the JBI Reviewers’ Manual recommendations, with a search in the MEDLINE, CINAHL, Web of Science, Scopus databases and in the Virtual Health Library portal, through articles published between 2016 and December 2021.

**Results:**

of the total 419 articles found, nine were selected for analysis. The main care measures identified were changing dressings and types of coverage, vital sign control, non-pharmacological techniques for pain relief and opioid reduction.

**Conclusions:**

the complexity of burn care requires constant updating by the nursing team. Keeping it prepared to carry out the best nursing care practices for burn patients will promote adequate care, patient recovery and reduction of possible harm.

## RESUMO

**Objetivos:**

identificar na literatura e sintetizar os cuidados de enfermagem em ambiente hospitalar direcionados aos pacientes que sofreram queimaduras.

**Métodos:**

revisão de escopo, segundo recomendações do JBI *Reviewers’ Manual*, com busca nas bases MEDLINE, CINAHL, *Web of Science*, Scopus e no portal Biblioteca Virtual em Saúde, através de artigos publicados entre 2016 e dezembro 2021.

**Resultados:**

do total 419 artigos encontrados, nove foram selecionados para análise. Os principais cuidados identificados foram troca de curativos e tipos de coberturas, controle dos sinais vitais, técnicas não farmacológicas para atenuação da dor e redução de opioides.

**Conclusões:**

a complexidade do cuidado em queimaduras requer constantes atualizações da equipe de enfermagem. Mantê-la preparada para a realização das melhores práticas de cuidado de enfermagem aos pacientes queimados promoverá a assistência adequada, recuperação do paciente e redução de possíveis danos.

## RESUMEN

**Objetivos:**

identificar en la literatura y resumir los cuidados de enfermería en ambiente hospitalario dirigidos a pacientes que sufrieron quemaduras.

**Métodos:**

revisión de alcance, según las recomendaciones del JBI *Reviewers’ Manual*, con búsqueda en MEDLINE, CINAHL, *Web of Science*, Scopus y en el portal Biblioteca Virtual en Salud, a través de artículos publicados entre 2016 y diciembre de 2021.

**Resultados:**

del total de 419 artículos encontrados, nueve fueron seleccionados para el análisis. Las principales medidas de atención identificadas fueron cambio de apósitos y tipos de cobertura, control de signos vitales, técnicas no farmacológicas para el alivio del dolor y reducción de opioides.

**Conclusiones:**

la complejidad del cuidado de quemados exige una constante actualización por parte del equipo de enfermería. Mantenerlo preparado para llevar a cabo las mejores prácticas de atención de enfermería a los pacientes quemados promoverá la atención adecuada, la recuperación del paciente y la reducción de posibles daños.

## INTRODUCTION

Defined as injuries to organic tissues, caused by chemical, physical and biological agents that compromise body tissues and cause cell death^(1)^, burns are considered the fourth most common type of trauma in the world^(2)^. In developing countries, such as Brazil, they are considered a public health problem and one of the main causes of mortality and partial or permanent disability^(2)^.

The World Health Organization (WHO) states that annually about 130,000 people are affected by some type of burn. In Brazil, it is estimated that one million burn accidents occur per year^(3)^, of which 10% seek hospital care and, of these, about 40,000 are hospitalized in a serious condition, requiring a quick, precise and adequate approach to define treatment^(4)^. It is noteworthy that 2 to 3% of these patients will die directly or indirectly^(5)^.

Northern Brazil is the one that least notifies the numbers for statistics, but it is the one that suffers most from fires in the country^(6)^. According to DATASUS, from June 2021 to June 2022, 4,487 hospital procedures were performed for the treatment of burns, corrosion and frostbite (ICD-10 T30) in the region. These data are relevant and show that, although most burned patients do not seek health services, and among those who do, there is underreporting, the number of consultations is still significant, which justifies the need for constant investment by the health system in prevention actions and appropriate management for this profile of patients^(7)^.

Despite advances in therapeutic procedures, burns in adult patients are considered a devastating aggression to human beings^(8)^, as they are responsible for physical and psychological sequel resulting from the emotional impact, causing changes in life and social limitations^(9)^. In this regard, with a view to minimizing damage, care should be initiated by assessing burned patients’ vital conditions and estimating the affected area. Then, injury assessment should be carried out, which aims to support and guide the actions to be taken^(10)^. Patients’ prognosis is directly associated with the extent of the affected body surface, the affected body region and injury depth^(11)^.

To describe the severity and prognosis of a burn, it is necessary to delineate issues such as the causal agent, depth and extent of a burned body surface area (BSA), which, once determined, will support the choice of treatment^(12)^. The “Rule of Nines” is often used to calculate the BSA, consisting of dividing the body surface of adults into multiples of nine, as it is more accurate in relation to body proportion and age^(13)^.

Knowing injury characteristics defines the initial treatment, however the therapeutic process results in long periods of hospitalization and rehabilitation treatments, with clinical and surgical procedures that impact patients’ quality of life. Therefore, the methods used for treatment must be directed by health services based on scientific evidence, aiming to minimize the sequelae caused and promote, when possible, patients’ total recovery^(14-15)^.

The nursing team is composed of professionals who work on the front line during health care provision. Therefore, the planning, monitoring and prevention of diseases secondary to burns are essential activities and aim at sustaining vital function, helping with rehabilitation, promoting quality of life and contributing to better clinical outcomes^(16)^. Moreover, these professionals must strive to minimize length of hospital stay, complications, sequel and the morbidity and mortality rate^(17)^.

Thus, knowing the necessary nursing care for adult patients who have suffered burns is essential to devise strategies and reorganize the execution of nursing work, in order to minimize sequelae and other effects. The lack of adequate and specific care can lead to greater complications and longer hospital stays, showing that qualified nursing teams are essential for the recovery of patients who have suffered burns^(18)^.

## OBJECTIVES

To identify in the literature and summarize the nursing care in a hospital environment directed to patients who suffered burns.

## METHODS

### Ethical aspects

Ethical assessment was not necessary, since the material used is in the public domain and does not involve human beings.

### Study design

This is a scoping review structured according to the recommendations of the international PRISMA guide^(19)^ and the method proposed in the JBI^(20)^. This technique has been widely used in the area of health sciences, with the aim of synthesizing and disseminating the results of studies on a subject^(21)^. Its main objective is to map concepts that support a given area of knowledge, examine the extent, scope and nature of the investigation, summarize and disseminate research data and identify gaps in existing research^(22)^.

### Search strategy and data source

In order to fulfill the steps of the proposed study, the objective of the study and the research question were listed, according to the PCC mnemonic combination: P (Population) - patients who suffered burns; C (Concept) - nursing care; C (Context) - hospital environment. Based on these definitions, the aim of the study was to identify in the literature and summarize nursing care in a hospital environment directed at adult patients who suffered burns, with the following guiding question: what is the nursing care in the hospital environment, directed to adult patients who suffered burns, available in the literature?

The descriptors and searches in the databases were selected from September to January 2022. It began with the search for the most used descriptors in searches, on the subject, contained in MEDLINE (via PubMed) and CINAHL (via EBSCO), followed by a broader search, using the same keywords and search terms in the databases Web of Science, Science Direct (via the Scopus platform), Cochrane Database of Systematic Reviews and the Virtual Health Library (VHL) portal.

The search strategy was developed together with a professional librarian from the *Universidade Federal do Paraná* (UFPR). The Health Sciences Descriptors and Medical Subject Headings (DeCS/MESH) were “Nursing”, “Nursing Care” and “Burns”, in English, Spanish and Portuguese. In the Scopus platform, the descriptors “Protocol” and “Validation Study” were included in the three languages.

The descriptors “Validation Study” and “Protocol” were included with the purpose of expanding the review, aiming at the search for scientific articles that bring validated care protocols, based on the best scientific evidence available and on local conditions, professional experience and client preferences.

Data collection took place between December 2021 and January 2022. Chart 1 fully presents the search strategies in each data source.

**Chart 1:** Search strategy, Porto Velho, Rondônia, Brazil, 2022

Data source	Search strategy adopted
VHL	(“Nursing” OR “*Enfermería*” OR “*Enfermagem*”) AND (“Nursing Care” OR “*Atención de Enfermeria*” OR “*Cuidados de Enfermagem*”) AND (“Burns” OR “*Quemaduras*” OR “*Queimaduras*”)
CINAHL	
MEDLINE	
COCHRANE	
Scopus	(“Burns” OR “*Quemaduras*” OR “*Queimaduras*) AND (Protocol) AND (“Nursing” OR “*Enfermería*” OR “*Enfermagem*”) AND (“Nursing Care” OR “*Atención de Enfermeria*” OR “*Cuidados de Enfermagem*”) AND (“Validation Study”)

*VHL - Virtual Health Library.*

**Chart 2:** Characterization of studies according to code S1 to S9, year and place of publication, method, level of evidence and degree of recommendation, objectives and results related to the subject of the study, Porto Velho, Rondônia, Brazil, 2021

ID	YEAR OF PUBLICATION/COUNTRY/ DATA PLATFORM	STUDY DESIGN/LoE/DEGREE OF RECOMMENDATION	OBJECTIVE	RELATED RESULTS
S1^(26)^	2017/Brazil/VHL	Case study/3B/B	Report the evolution of healing in a patient with second-degree burns treated with 0.2% hyaluronic acid (HA) and biocellulose film.	Isolation of nerve endings from the skin promoted by the dressing and reduced frequency of change, minimizing the occurrence of interventions that result in pain.
S2^(29)^	2018/United Kingdom/VHL	Case study/3B/B	Use biosynthetic dressing on superficial dermal burns in the pubic region.	Dressing use can play a significant role in reducing wound infection and desiccation rates.
S3^(27)^	2017/Spain/VHL	Case study/4/C	Present the therapeutic approach that can help other professionals to know the importance of the initial treatment of these injuries to prevent and/or solve future complications.	Burn treatment in three stages: wound cleaning and decongestion of phlebolymphedema, tissue repair and epithelialization and remodeling.
S4^(30)^	2018/Brazil/VHL	Case study/4/C	Implement the NP in the context of care of a burned patient assisted in a public health institution.	The following were listed as priorities: ineffective breathing pattern, risk of infection and impaired skin integrity, and the planning and definition of goals, interventions and activities to be implemented for further assessment were carried out. The NP implementation enables the development of quality assistance, based on scientific knowledge.
S5^(25)^	2016/USA/VHL	Descriptive study/2B/B	Measure the prevalence of acute neuropathic pain in patients with acute burns and the demographic and clinical characteristics.	Routine assessment of pain during the acute phase of injury with the aim of identifying patients who require further assessment, treatment and management.
S6^(33)^	2020/Israel/PubMed	Case study/4 /C	Assess the effectiveness of applying bromelain-based selective enzymatic debridement.	The safe and effective use of the agent, considered an effective treatment modality.
S7^(31)^	2018/USA/PubMed	Cross-sectional study/2C/B	Assess the effect of music therapy on pain, anxiety, opioid use and hemodynamic variables during burn dressing change.	Reduction in anxiety scores before dressing changes compared to daily dressing changes without the intervention.
S8^(28)^	2017/USA/PubMed	Randomized clinical trial/1B/A	Observe the effect of a rehabilitation intervention on the general health status of patients with hand burns.	Application of the Burn-Specific Abbreviated Health Scale (BSHS-A) and the rehabilitation intervention model with three phases: acute (T1), convalescence (T2) and before discharge (T3).
S9^(32)^	2019/USA/CINAHL	Randomized clinical trial/1B/A	Compare the effects of aromatherapy by inhaling apricot rose scent and the Benson relaxation technique on painful anxiety in burn patients.	After painful interventions, such as dressing changes, significant differences in pain anxiety were reported.

*USA - United States of America; S - study; LoE - level of evidence; ID - identification; VHL - Virtual Health Library; NP - Nursing Process.*

### Data collection, organization and analysis

The complete articles were considered from the reading of titles by two independent researchers and, after reading abstracts, a pre-selection of those that fit the inclusion criteria of this review was carried out. In case of possible doubts or disagreements with the analysis of abstracts and/or their relevance to the study, there would be the inclusion of a third researcher.

Study refinement was based on eligibility criteria. Included were studies carried out in adult patients who suffered burns, with themes involving nursing, medicine and health, available in full for free, in Portuguese, English, Spanish. Studies that addressed patient care outside the hospital environment, pediatric patients, involving other areas of activity, theses, dissertations, reviews and non-indexed publications were excluded. The period from January 2016 to December 2021 was defined as a time frame, in order to cover current nursing care on the theme.

Data were extracted, organized and characterized in Microsoft Office Excel^®^ spreadsheets in the following order: authors, year of publication, place of publication and data source, objectives, methodology and related results. Subsequently, they were assessed for the level of evidence (LoE) and degree of recommendation, according to the Oxford Center Evidence-Based Medicine^(23)^. Then, they were analyzed using simple descriptive statistics (relative and absolute), presented in a chart and discussed with support from the literature. Data were obtained without disagreement between reviewers, who did not consider the inclusion of the third researcher or the need to contact the primary authors about the data.

## RESULTS

**Figure 1:**
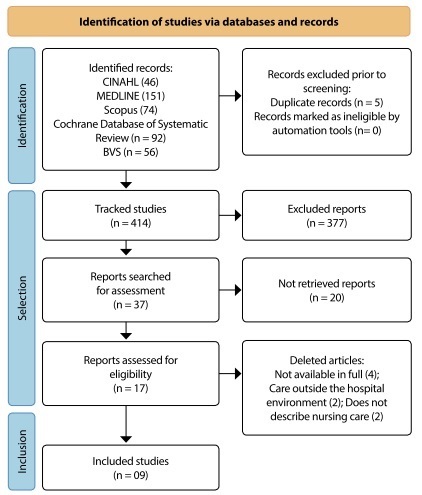
PRISMA diagram referring to the study selection process, 2022

Based on the analysis of 419 studies identified in the initial search, nine dealt with the topic addressed and corresponded to the final sample. Figure 1 specifies the results of the analysis steps, following the PRISMA Flow Diagram model^(24)^.

Of the nine selected studies, one^(25)^ was published in 2016 (11.1%), three^(26-28)^ in 2017 (33.3%), three^(29-31)^ in 2018 (33.3%), one^(32)^, in 2019 (11.1%) and one^(33)^, in 2020 (11.1%). The countries with the highest number of publications were the United States of America (USA)^(25,28,31-32)^, with four studies (44.4%), Brazil^(26,30)^, with two (22.2%), followed by the United Kingdom^(29)^, Spain^(27)^ and Israel^(33)^, with one each (33.3%).

Regarding the methodology used by the studies, five (55.5%) are case studies^(26-27,29-30,33)^, two (22.2%), randomized clinical studies^(28,32)^, one (11.1%), descriptive^(25)^ and one (11.1%), cross-sectional^(31)^. In the case of LoE, there was variation between 1B and 4, and the degree of recommendation, between A and C.

Analyzing LoE and degree of recommendation of selected studies, a higher frequency of LoE 3B^(26,29)^ and 4^(27,30)^ is observed, with a total of two studies in each (44.4%) of the sample. Two studies show LoE 1B^(28,33)^ (22.2%). With one study each (13.1%), two are levels 2B^(25)^ and 2C^(31)^. Regarding the degree of recommendation, two are degree A^(28,32)^ (22.2%), four are degree B^(25-26,29,31)^ (44.4%) and three are degree C^(27,30,33)^ (33.3%).

Regarding the objectives of the selected studies: one^(30)^ (11.1%) study highlights the importance of implementing the Nursing Process; four^(26-27,29-30)^ (44.4%) reinforce wound care and the importance of changing dressings and types of coverage for wound healing phases; two^(25,28)^ (22.2%) emphasize pain control assessment through scales and analgesia; and two^(31,33)^ (22.2%) emphasize non-pharmacological pain mitigation techniques to reduce the use of analgesia, such as music therapy, aromatherapy and relaxation techniques.

Among the results related to nursing care for patients who suffered burns, it was possible to observe that four^(26-27,29,33)^ (44.4%) assessed care for burn injuries, three^(25,28,30)^ (33.3%) analyze the interventions during the care of patients who suffered burns and two^(31-32)^ (22.2%) demonstrate non-pharmacological techniques to reduce pain.

Nursing care directed to care for burn injuries reinforces that promoting the re-epithelialization of affected areas and minimizing the occurrence of scars is one of the main challenges faced by the nursing team^(26)^. Appropriate initial management of the burn, correct diagnosis, choice of ideal coverage, providing rapid healing^(27)^, in addition to avoiding constant dressing changes, allow for a correct approach to injury and provide greater comfort and dignity to patients^(29,33)^.

It is recommended, as nursing interventions, that the team directs care, aiming at the well-being and improvement of patients who have suffered burns. Nurses must identify nursing diagnoses, plan and implement their actions, resulting in quality care^(30)^. Reassessing their actions, especially those aimed at patients’ pain during the wound healing phases, applying pain assessment scales and seeking treatment strategies, can directly affect patients’ psychological, social and functional rehabilitation^(25,32)^.

Pain and anxiety are present throughout the burn healing phases, and inadequate pain control, especially in the acute phase, can have a greater negative outcome in patients^(25)^. Non-pharmacological forms of treatment^(31-32)^, such as pain reduction techniques, help to reduce anxiety, pain and use of opioids during burn dressing changes.

## DISCUSSION

The studies that make up this review are those that contain information on the subject studied and present different nursing care aimed at adult patients who have suffered burns. Burns are harmful wounds and their characteristics are associated with the effects caused by injuries on body image and the implications of these in social circumstances. Associated with the complexity during the care provided to burned patients, nursing professionals require a set of skills to provide optimal and safe care.

Although burns mainly affect people in developing countries, such as Brazil, the USA and England were the precursor countries in the creation of services to improve treatment, called Burn Care Units (BTU), with the function of improving the indices in the care of patients who suffered burns^(34)^. This pioneering spirit may be related to the predominance of US publications among the studies in this review.

Although scientific and technological advances and the prognosis of burns have improved considerably in recent years, the success of care depends on the advancement of knowledge and the specific approach of health teams^(35)^. To this end, conducting research with primary source data is essential for discovering new techniques and conducts. However, this review demonstrates that the topic has been little explored in recent years, since the analyzed studies, in their predominance, adopt methodologies of bibliographic research, leaving the current literature scarce and generating gaps in the clinical decision-making processes and potential consequences for the population, the health system and the economy.

The prevalence of bibliographic studies may be related to lack of knowledge and ability of professionals to carry out research and apply it through evidence-based practice, revealing another gap. It should be noted that, even if there is a strong degree of recommendation in research for a given conduct, it is essential to analyze its suitability for a specific reality^(36)^.

With regard to nursing care for patients who have suffered burns, nursing actions must be comprehensive, since, in addition to wound healing, there are several needs in the process of recovery from burn injuries, such as psychobiological (oxygen therapy, hydration and nutrition, elimination, sleep and rest, body and oral hygiene, cutaneous-mucous and physical integrity, mobility, regulation, pain perception and therapy), psychosocial (communication and learning and gregarious) and psycho-spiritual (religious and ethical) needs. These care actions must be applied in the immediate, intermediate and late stages of burns, based on the care, educational and managerial contexts, using clinical reasoning for the elaboration and organization of nursing care^(33,37)^.

The importance of applying scales to assess the health status of patients who have suffered burns is observed. They are intended to measure the results of treatments and the impact of sequelae caused by burns on the daily life of this population, in addition to identifying whether patients require a more in-depth reassessment, treatment and management. For such an assessment, in addition to the pain control scale, the Burn Specific Health Scale (BSHS-R) can be used, which has the function of assessing the health status of patients who have suffered burns. The studies^(31,38)^ that applied the scale reported improvement in the levels of physical and psychological function, in social relationships and in patients’ general health conditions after the rehabilitation intervention.

The use of dressing was also one of the nursing care found as a result. Due to their many benefits, dressings are considered a widely used measure in injuries of burn patients. In addition to preventing external infections and ensuring local temperature control, they contribute to absorption of fluids released by the wound and have a specific coverage area according to the injury’s aspect^(39)^.

In order to carry out better care practices for patients who have suffered burns, the nursing team must be prepared and updated on the subject. Therefore, having extensive knowledge about the physiognomy of injuries caused by burns helps in decision-making and in the elaboration of an intervention plan that promotes patient recovery, reducing possible damages, since the variability of treatment of an injury may be related, among others, to lack of training, professionals’ attitude and discontinuity of care^(30,40)^.

Among the various care provided by the nursing team to patients who have suffered burns, the most frequent are changing dressings, as they cause daily and significant pain and discomfort to patients^(37)^. Different types of dressings are available on the market, however, among the selected studies, it was shown that the safe and effective use of bromelain-based enzymatic debridement agents, beyond the recommended and suggested 48 hours, has satisfactory success rates in the injuries of patients who have suffered burns, being considered an effective treatment modality, including burns of late presentation and chronic wounds.

The choice of dressing and wound care cannot be an automatic procedure, but a ‘scientific exercise’, in which nurses must act consciously in order to apply measures that can facilitate the healing process. Therefore, in order to define the best type of coverage, a careful analysis of wound characteristics, patients’ clinical conditions and cost/benefit ratio must be taken into account^(33,38,41)^.

Of the analyzed studies, pain stood out as one of the main symptoms to be managed by nursing care for patients who suffered burns. Patients’ pain has a significant impact during their treatment, therefore, it is necessary to manage it properly, due to the biological, emotional and/or social consequences that it may have^(42)^.

Patients who have suffered burns experience intense pain during and after surgical and non-surgical interventions. The use of drugs, such as opioid analgesics, is the main and most effective form of pain management^(43)^. Opposing this result, studies^(32,44)^ show that the use of non-pharmacological techniques to reduce pain, such as music therapy, aromatherapy and relaxation techniques, will demonstrate significant results after their application. The authors pointed out that, after using these methods, a reduction in the use of opioids was also observed, so that nursing professionals can provide these interventions, helping to reduce pain before performing painful procedures.

### Study limitations

As a limitation, we highlight the non-inclusion of manuals, theses, dissertations and legislation from other countries. As this review study only addressed studies published from 2016 to December 2021, it may have restricted relevant articles, becoming a limitation in the survey of potentially important studies from a clinical point of view, despite the insistent search for data that corroborate with the best selection criteria in the databases and studies of several countries. Therefore, there is a need to develop studies with greater methodological impact, highlighting the need to develop more expressive research in the study area.

### Contributions to nursing, health or public policy

Identifying the care inherent to patients who have suffered burns is important to list methods and reorient the execution of nursing work, in order to minimize sequelae and their consequences, as they directly reflect on patient safety and quality of care, in addition to cost reduction and patient morbidity and mortality.

## CONCLUSIONS

Nursing care for adult patients who suffered burns identified in this review began with an assessment of their needs, given that, in order to base the implementation of best care practices, the nursing team must be prepared and updated on the subject. The complexity of care in this area requires constant updates from the team, therefore, it is necessary to maintain the permanent education routine to adapt the training of nursing professionals to the reality and use of products and services.

Having extensive knowledge about the pathophysiology of injuries and healing helps in decision-making and in the elaboration of an intervention plan that promotes patient recovery, reducing possible damages. Also, care related to pain control, wound cleaning, dressing changes and best nursing practices was evidenced, with safety promotion and training for updating.

From study analysis, it is observed that nursing assistance in the care process plays a fundamental role and that the team must have a specific and standardized approach to nursing care in pain relief and control and in the early detection of complications. In this way, it is expected that this study will help in the dissemination of the best evidence regarding nursing care for patients who have suffered burns, awakening interest in new productions, in order to address other phases of the nursing process, such as nursing diagnoses and nurses’ exclusive attributions and with a greater degree of academic expressiveness.
